# Association between cardiac biomarkers and LVEF after congenital heart disease surgery in infants

**DOI:** 10.3389/fped.2025.1693380

**Published:** 2025-11-12

**Authors:** Yuxiao Mo, Qing Feng, Xia Wang, Shuwen Feng, Cong Wei, Minghui Zou, Jinping Liu, Junwen Zheng, Dongchi Zhao

**Affiliations:** 1 Department of Pediatrics, Women and Children’s Hospital, Zhongnan Hospital, Wuhan University, Wuhan, China; 2 Department of Cardiovascular Surgery, Zhongnan Hospital, Wuhan University, Wuhan, China

**Keywords:** congenital heart disease, surgical intervention, cardiac biomarkers, LVEF, infants

## Abstract

**Objective:**

Cardiac biomarkers are widely used to evaluate postoperative myocardial injury, but studies on their relationship with cardiac function in pediatrics are limited. This study aimed to characterize the dynamic changes of cardiac biomarkers after congenital heart disease (CHD) surgery in patients aged 0–3 years and assess their correlation with cardiac function.

**Methods:**

We retrospectively enrolled 63 pediatric patients (0–3 years) who underwent CHD surgery at Zhongnan Hospital between January 2021 and June 2024. Biomarkers and left ventricular ejection fraction (LVEF) were measured preoperatively and postoperatively within 1 week. The associations between the biomarkers and LVEF were analyzed. Receiver operating characteristic (ROC) curves were used to assess the predictive accuracy of the biomarkers for cardiac dysfunction, composite complications, and early mortality.

**Results:**

Biomarker levels peaked on postoperative day (POD) 1 and declined to near-baseline levels within 1 week. LVEF decreased initially and then gradually recovered to near preoperative levels by POD 4–7. LVEF on POD 1 negatively correlated with the peak high-sensitivity troponin I (hs-TnI) and creatine kinase–MB (CK-MB) levels. The ROC analysis showed that hs-TnI and CK-MB had good predictive power for cardiac dysfunction (AUC = 0.818 and 0.828) and composite complications (AUC = 0.736 and 0.745), but limited value for early mortality (AUC = 0.665).

**Conclusion:**

Hs-TnI and CK-MB are key biomarkers that reflect the relationship between myocardial injury and cardiac function recovery after CHD surgery in pediatric patients aged 0–3 years. Their peak postoperative levels can predict cardiac function recovery and composite complications.

## Introduction

Congenital heart disease (CHD) is a disease caused by abnormal development of the heart structure during fetal life. The global birth prevalence of CHD is estimated to be approximately 9.1 per 1,000 live births (1). Severe uncorrected cardiac malformations frequently lead to hemodynamic disturbances, resulting in complications such as heart failure, pulmonary hypertension, shock, and multiple organ dysfunction and representing one of the major causes of early mortality in infants (2, 3). Therefore, timely surgical intervention to correct cardiac malformations is critical for preserving normal cardiac function (4).

However, cardiac surgery is often associated with varying degrees of myocardial injury and cardiac dysfunction. Procedures such as cardiopulmonary bypass (CPB) and aortic cross-clamping (ACC) induce ischemia-reperfusion injury, oxidative stress, and inflammatory activation, which collectively exacerbate myocardial damage and impair cardiac recovery (5–7), leading to the release of myocardial biomarkers into the circulation, including cardiac troponin (cTn), creatine kinase–MB (CK-MB), and myoglobin (Mb). These biomarkers are widely used for perioperative assessment of myocardial injury (8, 9).

Troponin is a heterotrimeric complex composed of three subunits: TnC, TnT, and TnI. It is expressed in both skeletal and cardiac muscles and plays a crucial structural role in excitation–contraction coupling (10). Cardiac troponin I and T (cTnI and cTnT) are encoded by distinct genes and differ completely from their skeletal muscle counterparts, skeletal troponin (sTnI and sTnT). In the postnatal period, cardiac tissue simultaneously expresses cTnI and sTnI for the first 9 months, after which cTnI becomes the predominant isoform in cardiomyocytes (11). cTn is a highly specific biomarker for myocardial injury (12). Over the past decade, high-sensitivity troponin (hs-Tn), including hs-TnI and hs-TnT, has become a cornerstone in the early diagnosis of myocardial damage. Compared to conventional assays, hs-Tn tests demonstrate markedly enhanced analytical sensitivity, enabling the detection of extremely low circulating cTn concentrations. This increased sensitivity allows for the identification of subtle myocardial injury that may otherwise go undetected by standard methods (13–15). While cardiac biomarkers are well-established tools for prognostic assessment after cardiac surgery in adults and older children (16–18), their clinical utility in younger pediatric populations—particularly infants—remains understudied. Furthermore, given infants’ reduced physiological tolerance to surgical stress, the correlation between postoperative biomarker levels and functional cardiac recovery remains poorly characterized.

This study aimed to examine the dynamic changes in cardiac biomarkers and assess the association between postoperative hs-TnI, CK-MB, and Mb levels and cardiac functional recovery in pediatric patients after CHD surgery.

## Methods

### Study design

This single-center retrospective study analyzed clinical data from pediatric patients with CHD who underwent corrective cardiac surgery at Zhongnan Hospital of Wuhan University between January 2021 and June 2024.

### Ethics statement

The study protocol received ethical approval from the Institutional Review Board of Zhongnan Hospital, Wuhan University (Approval No. 2025007K). Due to its retrospective design, the requirement for informed consent was waived. All the procedures complied with the ethical principles outlined in the Declaration of Helsinki. To ensure participant confidentiality, all collected data were anonymized and stripped of any personally identifiable information. Strict protocols were implemented for data collection, secure storage, and analysis to maintain patient privacy throughout the research process.

### Inclusion and exclusion criteria

This study enrolled pediatric patients aged 0–3 years with confirmed CHD who underwent corrective cardiac surgery. Surgical indications for large atrial septal defect (ASD), ventricular septal defect (VSD), or patent ductus arteriosus (PDA) included (1) hemodynamically significant left-to-right shunt [pulmonary-to-systemic blood flow ratio (Qp/Qs) ≥1.5], (2) pulmonary hypertension, (3) left ventricular volume overload, (4) impaired cardiac function, or (5) recurrent respiratory infections. In infants under 3 months, VSD closure was performed for large perimembranous defects accompanied by severe heart failure or recurrent pulmonary infections refractory to medical management.

The exclusion criteria comprised (1) genetic or metabolic disorders, (2) major extracardiac malformations, (3) severe renal dysfunction [eGFR (estimated glomerular filtration rate) <30 mL/min/1.73 m^2^], and (4) prior heart transplantation or catheter-based interventions.

All cardiac diagnoses were established and categorized in accordance with the International Classification of Diseases, 10th Revision (ICD-10) diagnostic criteria (19, 20). Surgical interventions and postoperative care were uniformly administered following our institution's standardized clinical protocols. Following surgery, patients were routinely admitted to the pediatric intensive care unit for comprehensive monitoring and specialized postoperative management.

### Data collection

This single-center retrospective study extracted clinical data from the electronic medical records of Zhongnan Hospital, Wuhan University. The collected variables included baseline characteristics, preoperative parameters, intraoperative data, and postoperative observations. The baseline data consisted of gender, date of birth, body weight, procedure date, and CHD classification. The preoperative parameters covered history of cardiac dysfunction, left ventricular ejection fraction (LVEF), and concentrations of hs-TnI, CK-MB, and Mb. The intraoperative data included the surgical approach and the duration of CPB. The postoperative parameters encompassed the duration of vasopressor use, mechanical ventilation time, total hospital stay, serial echocardiographic assessments of LVEF, and biomarker levels (hs-TnI, CK-MB, and Mb) recorded within 1 week post-surgery. Additional data included electrocardiogram reports; chest imaging (x-ray or CT); laboratory tests, such as blood cultures and inflammatory markers; operative notes; dialysis records; and clinician-documented observations during hospitalization.

### Cardiac surgery risk scoring

The Aristotle Basic Complexity Score (ABCS) was employed to objectively assess the technical difficulty and procedural complexity of the CHD operations (21). This validated system classifies surgical complexity into four levels based on anticipated mortality, postoperative morbidity risk, and technical challenges: Level 1 (1.5–5.9), Level 2 (6.0–7.9), Level 3 (8.0–9.9), and Level 4 (10.0–15.0). Higher scores correspond to greater surgical complexity and risk.

### Blood sample collection and biomarker analysis

Peripheral venous blood (3 mL) was collected at perioperative time points according to clinical indications. Samples were transferred to lithium heparin tubes and processed within 2 h by centrifugation at 3,000 × *g* for 15 min at 4°C. Plasma was aliquoted and stored at −80°C until batch analysis.

Hs-TnI and Mb were measured using the ARCHITECT i2000SR system (Abbott Laboratories, Abbott Park, IL, USA) with matched STAT reagent kits (hs-TnI: #3P25; Mb: #2K43), based on chemiluminescent microparticle immunoassay technology [hs-TnI range: 4.0–10.0 pg/mL, limit of quantification (LoQ) ≤10 pg/mL; Mb sensitivity ≤1.0 ng/mL, range: 2.7–1,200 ng/mL]. CK-MB was assayed on the AU5800 analyzer (Beckman Coulter, Brea, CA, USA) with proprietary reagents (Ningbo Ruiyuan Biotechnology Co., Ltd., Ningbo, China) using the immunoinhibition method (range: 0–200 U/L; intra-assay CV <6%). The reference ranges were as follows: hs-TnI, 0–26.2 pg/mL; CK-MB, 0–6.6 ng/mL; Mb, 0–140.1 ng/mL.

### Postoperative outcomes assessment

Postoperative cardiac function was systematically assessed using transthoracic echocardiography. Left ventricular systolic function was classified as follows: preserved function: LVEF >50%; impaired function: LVEF ≤50% (22, 23). The composite complications included severe arrhythmias requiring intervention, acute heart failure, low cardiac output syndrome, cardiac tamponade necessitating reoperation, sepsis/septic shock, pneumothorax, pulmonary hemorrhage, and acute kidney injury requiring renal replacement therapy.

The patient outcomes were classified as survival or early mortality (death within 30 days postoperatively).

### Statistical analysis

All statistical analyses were performed using R version 4.5.1 (R Foundation for Statistical Computing, Vienna, Austria). Continuous variables were expressed as median with interquartile range (IQR) or mean ± standard deviation (SD), as appropriate. Comparisons between two groups were conducted using the independent-samples *t*-test or the Mann–Whitney *U*-test. For comparisons among more than two independent groups, one-way analysis of variance (ANOVA) or the Kruskal–Wallis test was used. For repeated measures across multiple time points, the Friedman test was applied; if significant differences were detected, pairwise comparisons were performed using Bonferroni or Dunn's correction. Correlation analyses were conducted using Pearson or Spearman rank correlation coefficients, depending on data distribution. Receiver operating characteristic (ROC) curve analysis was used to assess diagnostic performance, and the area under the curve (AUC) was calculated. *P-*value <0.05 was considered statistically significant.

## Results

### Study population characteristics

A total of 63 pediatric patients who met the inclusion criteria were enrolled in this study (Table 1). The cohort consisted of 25 male (39.7%) and 38 female (60.3%) patients, with a male-to-female ratio of approximately 1:1.5. The median age at the time of surgical intervention was 4.1 months (IQR: 1.2–8.6 months). Among the patients, 23 (36.5%) were infants aged 0–3 months, including 10 neonates (<28 days, 15.9%). Older infants (3–12 months) accounted for 29 patients (46.0%), and toddlers (1–3 years) comprised 11 patients (17.5%).

**Table 1:** Baseline demographics of the patients with CHD.

Variable	Whole cohort (*n* = 63)	Survivors (*n* = 58)	Non-survivors (*n* = 5)	*P*-value
Age (months)	4.1 (1.2–8.6)	4.5 (1.7–9.3)	1.1 (0.3–3.3)	0.029
<3 months, *n* (%)	23 (36.5%)	19 (32.8%)	4 (80.0%)	
3 months–1 year, *n* (%)	29 (46.0%)	28 (48.3%)	1 (20.0%)	
1–3 years, *n* (%)	11 (17.5%)	11 (19.0%)	0 (0.0%)	
Weight (kg)	5.5 (3.6–8.5)	5.6 (3.6–8.5)	2.3 (2.1–5.1)	0.011
Gender, *n* (%)				
Female	38 (60.3%)	36 (62.1%)	2 (40.0%)	0.623
Male	25 (39.7%)	22 (37.9%)	3 (60.0%)	
Main cardiovascular anomalies, *n* (%)				
VSD	36 (57.1%)	32 (55.2%)	4 (80.0%)	0.278
ASD	8 (12.7%)	8 (13.8%)	0 (0.0%)	
PDA	6 (9.5%)	6 (10.3%)	0 (0.0%)	
Others	13 (20.7%)	12 (20.7%)	1 (20.0%)	
ABCS levels, *n* (%)				
Level 1	16 (25.4%)	16 (27.6%)	0 (0.0%)	0.299
Level 2	33 (52.4%)	29 (50.0%)	4 (80.0%)	
Level 3	12 (19.0%)	11 (19.0%)	1 (20.0%)	
Level 4	2 (3.2%)	2 (3.4%)	0 (0.0%)	
Surgery on CPB, *n* (%)	54 (85.7%)	49 (84.5%)	5 (100%)	0.549
CPB duration (min)	96.0 (74.5–120.0)	97.0 (74.5–120.0)	90.5 (76.0–123.8)	0.469
Cardiotomy, *n* (%)	51 (81.0%)	46 (79.3%)	5 (100.0%)	0.573
Length of stay (days)	16 (13–21)	16 (13–21)	13 (7–29)	0.704
Duration of MV (days)	2 (1–5)	2 (1–5)	1 (0–6)	0.470
Duration of vasopressor (days)	6 (3–8)	6 (4–8)	5 (2–8)	0.852
Peak hs-TnI (pg/mL)	11,912.5 (6,478.0–17,983.2)	9,627.8 (5,131.1–17,953.4)	28,524.0 (13,101.7–44,918.1)	0.043
Peak CK-MB level (ng/mL)	120.7 (85.0–212.2)	106.5 (72.5–177.6)	137.9 (93.6–262.5)	0.381
Peak Mb level (ng/mL)	551.9 (324.4–917.6)	453.8 (321.6–724.4)	936.2 (599.6–1,138.7)	0.058
POD 1 LVEF (%)	60.0 (56.0–65.0)	62.0 (58.8–65.0)	40.0 (15.0–63.5)	0.119

VSD, ventricular septal defect; ASD, atrial septal defect; PDA, patent ductus arteriosus; ABCS, Aristotle Basic Complexity Score; CPB, cardiopulmonary bypass; MV, mechanical ventilation; hs-TnI, high-sensitivity troponin I; CK-MB, creatine kinase–MB isoenzyme; LVEF, left ventricular ejection fraction; POD, postoperative day.

According to the classification of cardiac malformations, 36 patients (57.1%) had VSD, 8 (12.7%) had ASD, and 6 (9.5%) had PDA. Coarctation of the aorta (4, 6.3%), tetralogy of Fallot (3 cases, 4.8%), atrioventricular septal defect (2, 3.1%), complete transposition of the great arteries (1, 1.6%), total anomalous pulmonary venous return (TAPVC; *n* = 1, 1.6%), congenital pulmonary valve stenosis (1, 1.6%), and subaortic membrane (1, 1.6%) were among the other complex congenital heart defects that were present in the remaining 13 patients (20.6%). In total, 54 (85.7%) of the 63 patients underwent surgery with CPB assistance. According to the ABCS classification, 16 patients (25.4%) underwent level 1 procedures, 33 (52.4%) underwent level 2 procedures, and 14 (22.2%) underwent level 3–4 procedures.

Five deaths (9.8%) occurred within 30 days after surgery, and detailed demographic, operative, and postoperative characteristics are presented in Supplementary Appendix 1. The non-survivors had a median age of 1.1 month (range: 3 days–5 months) and a median body weight of 2.3 kg (range: 2.0–5.8 kg), including four neonates. The underlying diagnoses included VSD (*n* = 4) and TAPVC (*n* = 1). Importantly, all of these patients underwent corrective surgery (VSD closure or TAPVC repair), and none received palliative surgery such as pulmonary artery banding. Three deaths occurred on the day of surgery, with peak postoperative hs-TnI levels exceeding 40,000 pg/mL. The primary causes of death were heart failure (*n* = 4) and multiple organ dysfunction syndrome (*n* = 1).

Overall, the median postoperative peak hs-TnI level in the non-survivors was 28,524.0 pg/mL (IQR: 13,101.7–44,918.1 pg/mL), significantly higher than that in the survivors (9,627.8 pg/mL, IQR: 5,131.1–17,953.4 pg/mL; *P* = 0.043). However, there were no significant differences between the two groups in peak CK-MB or Mb levels (*P* > 0.05).

### Age-related changes in cardiac biomarkers after surgery

Given that the expression of sTnI declines with age after birth and cTnI becomes predominant in infants older than 9 months, we analyzed hs-TnI levels before and after cardiac surgery in the patients stratified by age (Supplementary Appendix 2).

Preoperative hs-TnI levels were significantly higher in infants ≤9 months (19.1 pg/mL, IQR: 5.8–45.4 pg/mL) compared to those >9 months (1.6 pg/mL, IQR: 0.4–6.5 pg/mL) (*P* = 0.005), with the median value being approximately 10-fold higher in the younger group. However, the postoperative hs-TnI levels did not differ significantly between the two groups, with 11,807.1 pg/mL (IQR: 4,699.1–23,342.5 pg/mL) in the ≤9-month group and 11,105.4 pg/mL (IQR: 7,042.1–15,577.2 pg/mL) in the >9-month group (*P* = 0.593). To evaluate whether the difference in preoperative hs-TnI levels was related to baseline clinical severity, we compared preoperative LVEF, history of heart failure, ABCS, and the proportion of patients undergoing CPB between the two age groups (Supplementary Appendix 3). No significant differences were observed in preoperative LVEF (*P* = 0.261), ABCS (*P* = 0.338), or the proportion of patients receiving CPB (*P* = 0.587). However, the proportion of infants with a history of preoperative heart failure was significantly higher in those aged ≤9 months (25/48, 52.1%) compared to those older than 9 months (2/15, 13.3%; *P* = 0.008).

### Impact of surgical complexity on cardiac biomarkers

To evaluate the effect of surgical complexity on myocardial injury in infants, the patients were stratified into two groups based on ABCS, namely, the low-complexity group (levels 1–2, *n* = 49) and the high-complexity group (levels 3–4, *n* = 14). The proportion of patients that underwent CPB was not significantly different between the two groups [85.7% (42/49) vs. 64.3% (9/14); *P* = 0.157]. Postoperative peak cardiac biomarker levels, including hs-TnI, CK-MB, and Mb, were compared between the two groups (Supplementary Appendix 4). No statistically significant differences were observed in any of the biomarkers between the two groups (*P* > 0.05).

### Effect of surgical invasiveness on cardiac biomarker elevation

To further assess the impact of surgical invasiveness on myocardial injury, the patients were divided into two groups based on whether they received CPB or underwent an intracardiac operation. Patients who underwent cardiac chamber incisions with CPB were categorized as the CPB group, whereas those who received off-pump procedures were assigned to the Off-CPB group (Supplementary Appendix 4).

The postoperative peak hs-TnI level in the CPB group was significantly elevated, with a median of 11,912.5 pg/mL (IQR: 5,732.4–18,843.8 pg/mL), nearly 200-fold higher than that in the Off-CPB group [58.5 pg/mL (IQR: 48.1–174.0 pg/mL); *P* < 0.001]. The CPB group also had a significantly higher CK-MB level [120.4 ng/mL (IQR: 85.0–202.5 ng/mL)] than the Off-CPB group [20.8 ng/mL (IQR: 14.8–25.4 ng/mL); *P* = 0.002]. In contrast, there was no significant difference in Mb levels between the two groups (*P* = 0.599), with values of 478.3 ng/mL (IQR: 321.6–902.5 ng/mL) in the CPB group and 638.5 ng/mL (IQR: 378.8–870.2 ng/mL) in the Off-CPB group.

### Trends in cardiac biomarkers and LVEF following cardiac surgery

**Figure 1:**
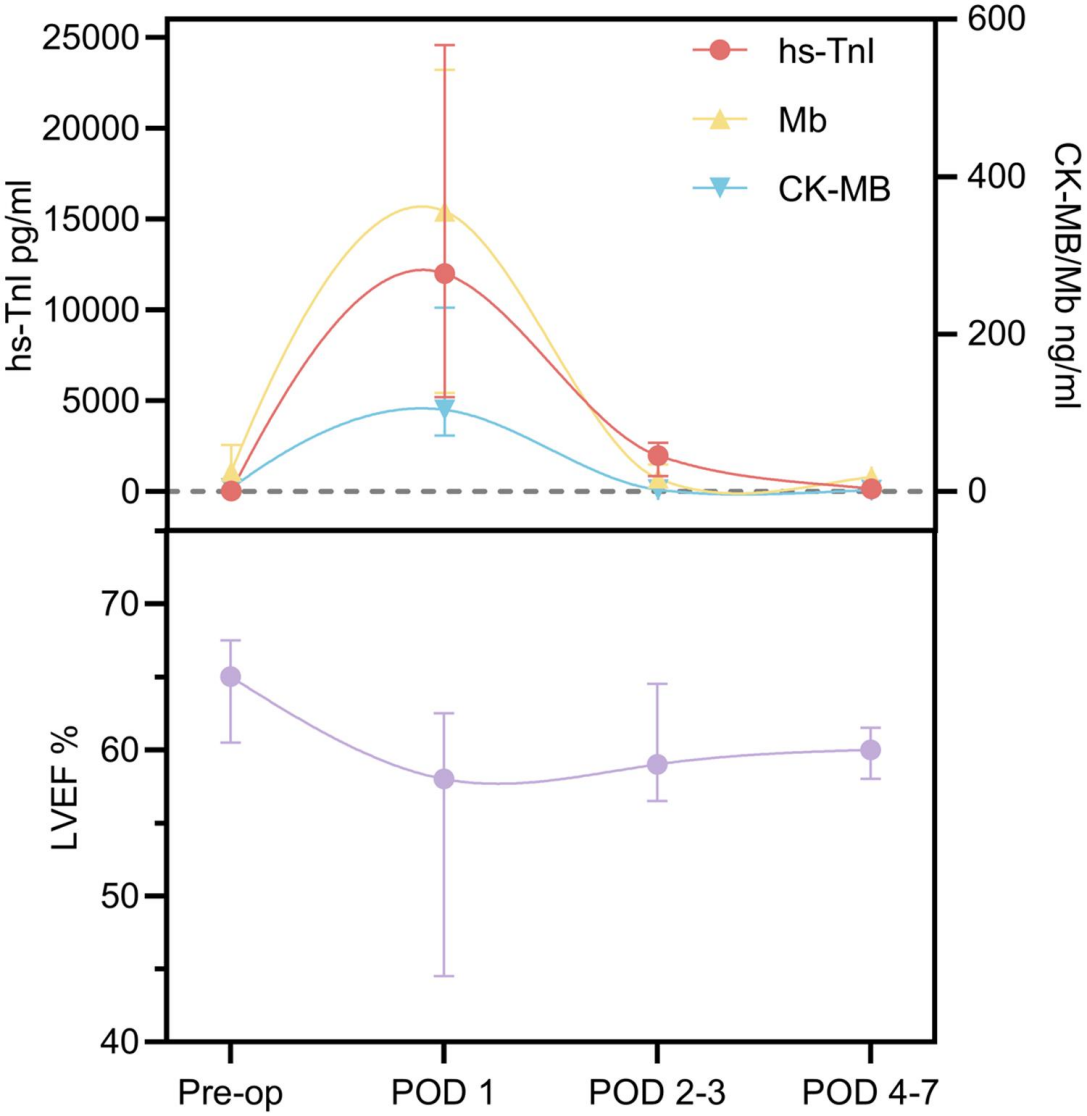
Cardiac biomarker and LVEF trends within 1 week after surgery. Pre-op, preoperative; POD, postoperative day; LVEF, left ventricular ejection fraction.

To evaluate the association between the postoperative myocardial biomarkers and cardiac function, the levels of hs-TnI, CK-MB, and Mb, along with LVEF, were periodically measured in the surviving patients who underwent cardiac chamber incisions on CPB (Figure 1).

LVEF declined significantly from a preoperative median of 65.0% (IQR: 60.5%–67.5%) to 58.0% (IQR: 44.5%–62.5%) on postoperative day (POD) 1, then gradually recovered to 59.0% (IQR: 56.5%–64.5%) on POD 2–3 and 60.0% (IQR: 58.0%–61.5%) on POD 4–7, returning to baseline levels. In contrast, the serum levels of hs-TnI, CK-MB, and Mb peaked on POD 1 and subsequently declined to near-baseline levels within 1 week. The Friedman test revealed significant time-dependent fluctuations in LVEF and all three cardiac biomarkers (hs-TnI, CK-MB, and Mb) (*P* < 0.05). Notably, the trends observed between the increased biomarker levels and changes in LVEF suggest a potential association between postoperative myocardial injury and transient declines in cardiac function. Further pairwise comparisons using Bonferroni correction (Supplementary Appendix 5) demonstrated that LVEF significantly decreased by approximately 10.8% on POD 1 compared to preoperative values (*P* < 0.001). No significant differences were observed between preoperative LVEF and the values on POD 2–3 and POD 4–7 (*P* > 0.05).

On POD 1, hs-TnI reached a peak of 12,000.7 pg/mL (IQR: 5,186.9–24,571.5 pg/mL), representing an approximate 670-fold increase compared to preoperative levels (*P* < 0.001), and then began to decline on POD 2–3 to 1,986.3 pg/mL, corresponding to an 83.7% decrease from the peak (*P* = 0.121), and further decreased to near baseline at 156.3 pg/mL (IQR: 71.1–361.8 pg/mL) on POD 4–7, representing a 98.7% reduction compared to the peak (*P* < 0.001).

The CK-MB levels increased 26-fold on POD 1 compared to preoperative values (*P* < 0.001), followed by a rapid decline to 2.3 ng/mL (IQR: 1.0–3.3 ng/mL) on POD 2–3, approaching baseline levels and representing a 93.7% decrease from the peak (*P* < 0.001). Similarly, the Mb levels significantly increased to 14 times the preoperative level on POD 1 (*P* = 0.044) and then decreased to near baseline by POD 2–3, reflecting a 95.3% reduction (*P* = 0.015).

### Correlation analysis between the myocardial biomarkers and perioperative parameters

**Figure 2:**
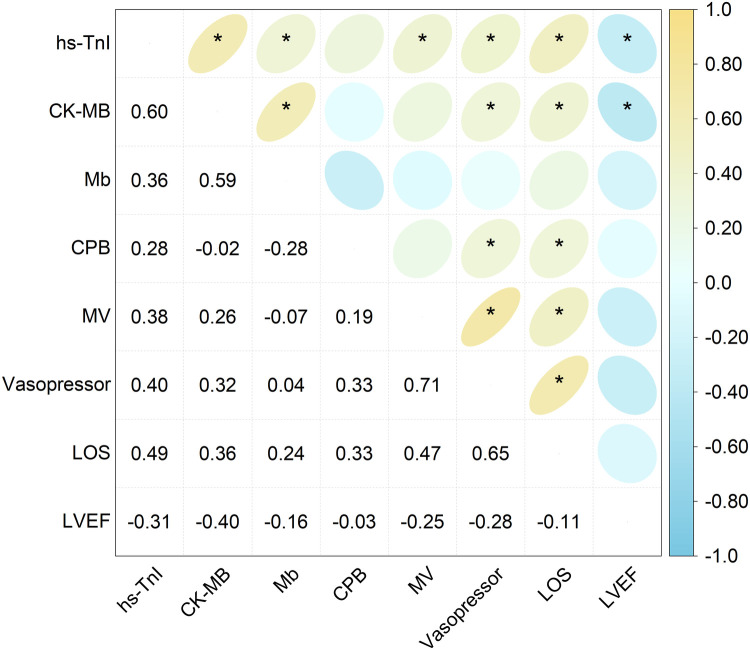
Correlation heatmap showing the relationship between the peak levels of the cardiac biomarkers and perioperative clinical parameters. The numerical values inside each grid denote the correlation coefficient (r). Asterisks indicate statistically significant correlations (*P < 0.05). hs-TnI, high-sensitivity troponin I; CK-MB, creatine kinase–MB isoenzyme; Mb, myoglobin; CPB, cardiopulmonary bypass; MV, mechanical ventilation; LOS, length of stay; LVEF, left ventricular ejection fraction.

The correlation analysis between the cardiac biomarkers and perioperative clinical parameters (Figure 2) demonstrated that the peak hs-TnI levels were significantly negatively correlated with POD 1 LVEF (*r* = −0.31, *P* < 0.05), and positively correlated with vasopressor duration (*r* = 0.40, *P* < 0.05), mechanical ventilation duration (*r* = 0.38, *P* < 0.05), and length of hospital stay (*r* = 0.49, *P* < 0.001). However, no significant correlations were observed between peak hs-TnI and CPB time.

Similarly, the peak CK-MB levels showed a significant negative correlation with LVEF (*r* = −0.40, *P* < 0.001) and positive correlations with vasopressor duration (*r* = 0.32, *P* < 0.05) and length of hospital stay (*r* = 0.36, *P* < 0.05), but were not correlated with CPB time or duration of mechanical ventilation. However, there were no discernible relationships between the peak Mb levels and any perioperative parameters.

### Evaluation of the diagnostic performance of the cardiac biomarkers by ROC curve analysis

**Figure 3:**
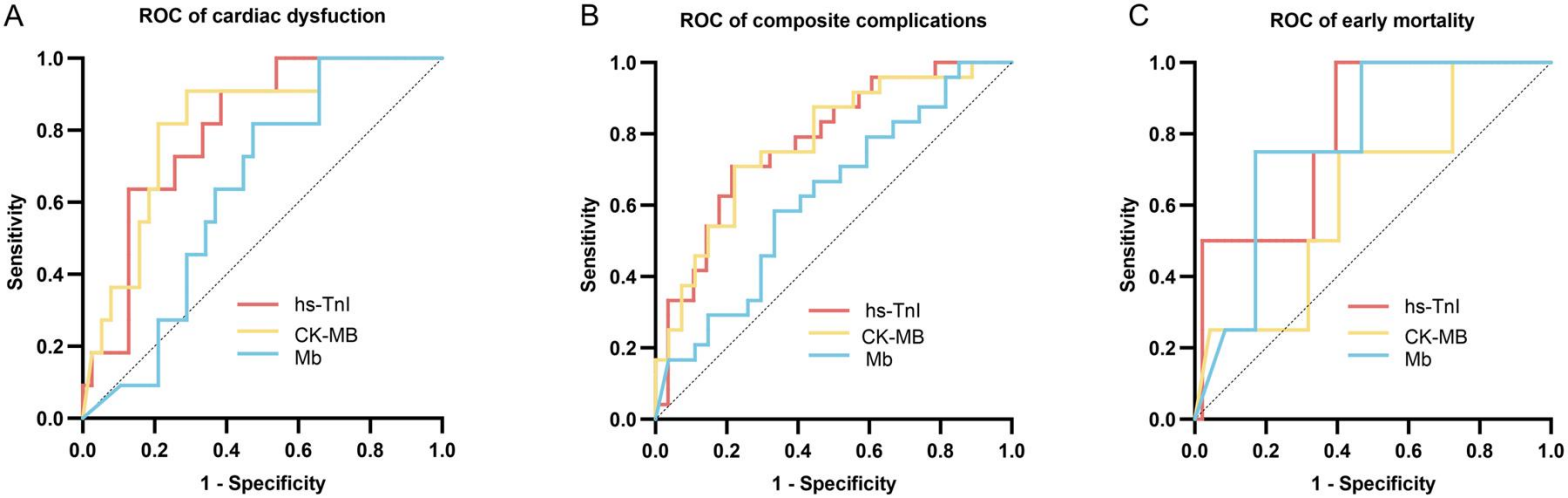
ROC curves evaluating the predictive performance of postoperative hs-TnI, CK-MB, and Mb levels. (**A**) ROC curves for predicting postoperative cardiac dysfunction. (**B**) ROC curves for predicting composite postoperative complications. (**C**) ROC curves for predicting early mortality. The curves demonstrate the discriminatory ability of each biomarker for the respective clinical outcomes. hs-TnI, high-sensitivity troponin I; CK-MB, creatine kinase–MB isoenzyme; Mb, myoglobin.

An ROC curve analysis was conducted to evaluate the diagnostic performance of postoperative peak levels of hs-TnI, CK-MB, and Mb in predicting clinical outcomes in pediatric patients with CHD. The sensitivity and specificity of these biomarkers in predicting postoperative cardiac dysfunction, composite complications, and early mortality were measured by ROC curves and AUC values (Figure 3; Table 2).

**Table 2:** Area under the curve and cutoff value (sensitivity, specificity) for each variable.

Cardiac biomarkers	AUC	Asymptotic 95% CI		Cutoff value (sensitivity, specificity)
		Lower limit	Upper limit	
Variable in the ROC curve for cardiac dysfunction				
hs-TnI (pg/mL)	0.818	0.693	0.943	11,301.85 (0.909, 0.632)
CK-MB (ng/mL)	0.828	0.700	0.956	123.15 (0.909, 0.737)
Mb (ng/mL)	0.646	0.491	0.801	437.75 (0.818, 0.553)
Variable in the ROC curve for composite complications				
hs-TnI (pg/mL)	0.736	0.598	0.847	12,145.05 (0.667, 0.778)
CK-MB (ng/mL)	0.745	0.609	0.882	120.55 (0.667, 0.778)
Mb (ng/mL)	0.596	0.439	0.753	549.85 (0.542, 0.667)
Variable in the ROC curve for early mortality				
hs-TnI (pg/mL)	0.665	0.229	0.370	12,145.05 (0.800, 0.609)
CK-MB (ng/mL)	0.509	0.206	0.812	123.15 (0.600, 0.609)
Mb (ng/mL)	0.635	0.331	0.938	916.45 (0.600, 0.826)

ROC, receiver operating characteristic; AUC, area under the curve; hs-TnI, high-sensitivity troponin I; CK-MB, creatine kinase–MB isoenzyme; Mb, myoglobin.

Postoperative peak hs-TnI (AUC = 0.818, 95% CI: 0.693–0.943) and CK-MB (AUC = 0.828, 95% CI: 0.700–0.956) demonstrated good predictive performance for postoperative cardiac dysfunction, whereas Mb showed relatively lower predictive ability (AUC = 0.646, 95% CI: 0.491–0.801). The optimal cutoff values determined by the Youden index were 11,301.85 pg/mL for hs-TnI, 123.15 ng/mL for CK-MB, and 437.75 ng/mL for Mb.

For prediction of composite postoperative complications, hs-TnI (AUC = 0.736, 95% CI: 0.598–0.847) and CK-MB (AUC = 0.745, 95% CI: 0.609–0.882) showed good predictive value, while the predictive performance of Mb was limited (AUC = 0.596, 95% CI: 0.439–0.753). The optimal cutoff values determined by the Youden index were 12,145.05 pg/mL for hs-TnI, 120.55 ng/mL for CK-MB, and 549.85 ng/mL for Mb.

All three biomarkers, namely, hs-TnI (AUC = 0.665, 95% CI: 0.229–0.370), CK-MB (AUC = 0.509, 95% CI: 0.206–0.821), and Mb (AUC = 0.635, 95% CI: 0.331–0.938), demonstrated suboptimal performance in predicting early postoperative mortality.

## Discussion

This study dynamically monitored cardiac biomarkers in pediatric patients who underwent CHD surgery to evaluate their association with cardiac function. According to our findings, LVEF dramatically decreased on POD 1 and then progressively recovered to preoperative levels, whereas the hs-TnI, CK-MB, and Mb serum levels peaked on POD 1 and returned to baseline within 1 week. Cardiac chamber incision under CPB significantly increased the biomarker levels. However, ABCS stratification had no significant impact on postoperative hs-TnI and CK-MB levels. The peak levels of hs-TnI and CK-MB were negatively correlated with LVEF, better reflecting cardiac function recovery. Both hs-TnI and CK-MB demonstrated high predictive accuracy for postoperative cardiac dysfunction and composite postoperative complications, but showed limited efficacy in predicting early postoperative mortality.

This study found no significant changes in CPB duration, postoperative CK-MB and Mb levels, or LVEF between the survivors and non-survivors, despite the fact that hs-TnI levels were considerably higher in the latter group. This suggests that early postoperative mortality may be more influenced by factors such as neonatal age and body weight. The analysis of hs-TnI levels in different age groups revealed that preoperative hs-TnI levels were significantly higher in infants aged ≤9 months compared to those aged >9 months. This may be related to the physiological characteristics of infants and the developmental expression pattern of troponin. Infants typically have a higher resting heart rate compared with older children and adults, with values commonly ranging from 120 to 160 bpm (24). In early life, cardiac output is primarily maintained through modulation of heart rate rather than stroke volume (25). Consequently, the infant myocardium operates at a relatively higher oxygen demand, which may render it more vulnerable to hypoxemia or subclinical injury under stress (26). During the first 9 months after birth, sTnI and cTnI are co-expressed in the heart, after which cardiac myocytes predominantly express cTnI (11).

Although the younger infants (≤9 months) demonstrated markedly higher baseline hs-TnI levels, reflecting increased preoperative myocardial vulnerability, the magnitude of postoperative hs-TnI release did not differ significantly from that of the older children. This finding appears to contradict the conventional assumption that infants are more susceptible to perioperative myocardial injury. Instead, it suggests that the extent of acute myocardial damage following corrective surgery is largely determined by intraoperative factors such as CPB duration and myocardial protection strategies (7, 27), rather than by age-related differences in myocardial development alone.

No significant differences were observed in postoperative cardiac biomarker levels among the different complexity groups, suggesting that surgical complexity has a limited influence on postoperative cardiac biomarker levels. However, the hs-TnI and CK-MB levels were significantly higher in the CPB group compared to the Off-CPB group. This is likely attributable to the direct myocardial injury caused by intracardiac surgery, compounded by ischemia-reperfusion injury and systemic inflammatory responses associated with CPB, all of which exacerbate myocardial damage (28–30).

Infants are particularly vulnerable to myocardial injury during heart surgery, which results in a significant release of cardiac biomarkers. In the analysis of the dynamic changes in the postoperative biomarker levels, hs-TnI, CK-MB, and Mb all peaked on POD 1 and declined rapidly, approaching baseline levels within 1 week. Notably, hs-TnI returned to physiological levels more slowly than CK-MB and Mb, suggesting that serial hs-TnI measurements may provide more reliable information on the extent and duration of myocardial injury in the early postoperative period. This pattern may reflect the robust myocardial repair mechanisms and efficient metabolic clearance capacity in infants (31). This trend is consistent with previous findings on troponin levels during the first postoperative week after pediatric heart transplantation (32). The postoperative changes in LVEF showed an opposite trend to the biomarkers, with LVEF decreasing on POD 1 followed by gradual recovery. This suggests that acute myocardial injury resulting from surgery may lead to a temporary reduction in left ventricular systolic function, whereas the subsequent normalization of myocardial biomarkers reflects concurrent myocardial repair and recovery of contractile function.

Although prolonged CPB duration is generally considered a potential contributor to postoperative myocardial injury, in our study, no significant correlations were found between CPB duration and postoperative peak levels of hs-TnI, CK-MB, or Mb. This finding suggests that the extent of myocardial injury in our cohort may be more strongly influenced by other perioperative factors—such as surgical complexity, myocardial protection strategy, and individual patient characteristics—rather than CPB duration alone.

The ROC analysis further confirmed that two of the biomarkers demonstrated high diagnostic accuracy in predicting postoperative cardiac dysfunction and composite complications. This finding is consistent with results reported in previous studies (33–36). A recent study in neonates with congenital heart disease reported that CK-MB levels were significantly higher in patients requiring surgical intervention and correlated with echocardiographic parameters of diastolic function, supporting the association between CK-MB elevation and myocardial dysfunction (37). In contrast, Mb showed no significant correlation with postoperative cardiac function or adverse outcomes, and its predictive efficacy in the ROC analysis was markedly lower. This discrepancy may be attributed to the shorter half-life and lower tissue specificity of Mb, which limit its ability to reflect the extent of myocardial injury in pediatric patients after cardiac surgery (38). In addition, none of the three biomarkers demonstrated significant predictive value for early postoperative mortality.

## Limitations

This study is a single-center retrospective analysis with a limited sample size, and the findings require validation in larger-scale studies. In addition, the assessment of postoperative cardiac function was limited. Although routine echocardiography was performed postoperatively, its accuracy in evaluating cardiac function is constrained, especially in patients who are administered vasoactive drugs. In such cases, some patients with impaired cardiac function may still present with normal LVEF. In addition, the majority of the patients were young infants, which limited the feasibility of frequent blood sampling due to safety concerns. Consequently, blood samples for hs-TnI, CK-MB, and Mb were collected according to clinical schedules rather than at strictly fixed intervals. Because of the variable timing and the influence of the biomarkers’ half-lives, the measured peak values may not reflect the true maximal concentrations. This limitation may affect the precision of perioperative myocardial injury assessment and the strength of the associations with clinical outcomes.

## Conclusion

This study systematically analyzed the dynamic changes in cardiac biomarkers and their relationship with cardiac function and prognosis in pediatric patients aged 0–3 years who underwent CHD surgery. All three biomarkers peaked on POD 1. The CK-MB and Mb levels declined to baseline by POD 2–3, while the hs-TnI levels decreased to near preoperative levels by POD 4–7. LVEF decreased on POD 1 and gradually recovered thereafter, showing an inverse trend compared to the biomarkers.

## Data Availability

The original contributions presented in the study are included in the article/Supplementary Material, further inquiries can be directed to the corresponding authors.
