# Supplementary material for: Association between cardiac biomarkers and LVEF after congenital heart disease surgery in infants
Source: Front Pediatr. 2025 Nov 12;13:1693380. doi: 10.3389/fped.2025.1693380 (PMC12647081; doi:10.3389/fped.2025.1693380)
Supplement: Supplementary file 1 [file Supplementaryfile1.docx]

Appendix 1 Demographic, operative, and postoperative data of died patients

| Deaths | Age | Gender | Weight(kg) | CHD | ABCS | CPB time(min) | POD 1  hs-TnI(pg/ml) | POD 1  Mb(ng/ml) | POD 1  CK-MB(ng/ml) | POD 1  LVEF(%) | survival days | Causes of death |
| --- | --- | --- | --- | --- | --- | --- | --- | --- | --- | --- | --- | --- |
| 1 | 3days | F | 2.23 | TAPVC | 9.0 | / | 45966.2 | 1200 | 52.9 | 68 | 1 | MODS |
| 2 | 1month | F | 2.33 | VSD | 6.0 | 96 | 41773.9 | 917.6 | 300.0 | 10 | 1 | HF |
| 3 | 17days | M | 2.00 | VSD | 6.0 | 85 | / | / | / | / | 1 | HF |
| 4 | 5months | M | 5.87 | VSD | 6.0 | 73 | 15274.1 | 493.6 | 150.1 | 50 | 1 | HF |
| 5 | 1month | M | 4.34 | VSD | 6.0 | 133 | 12377.6 | 954.8 | 125.6 | 30 | 9 | HF |

Abbreviations: CHD, congenital heart disease; ABCS, Aristotle basic complexity score; CPB, cardiopulmonary bypass; POD, postoperative day; hs-TnI, high-sensitivity troponin I; CK-MB, creatine kinase–MB isoenzyme; Mb, myoglobin; LVEF, left ventricular ejection fraction; F, female; M, male; TAPVC, total anomalous pulmonary venous connection; VSD, ventricular septal defect; MODS, multiple organ dysfunction syndrome; HF, heart failure.

Appendix 2. Comparison of preoperative and postoperative peak high-sensitivity troponin I (hs-TnI) levels between patients aged ≤9 months and >9 months. ** represents P < 0.01.


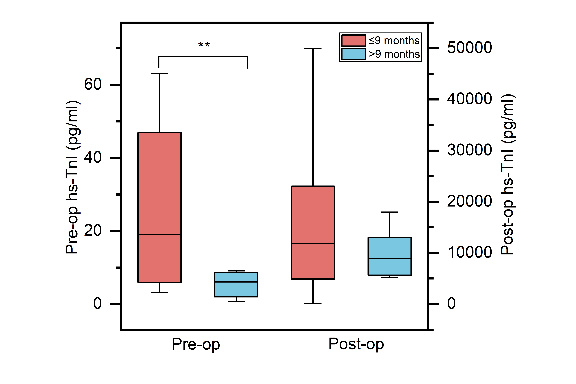


Abbreviations: Pre-op, preoperative; post-op, postoperative; hs-TnI, high-sensitivity troponin I.

Appendix 3 Comparison of preoperative clinical characteristics between patients ≤9 months and >9 months

|  | ≤9 months (n=48) | >9 months (n=15) | P value |
| --- | --- | --- | --- |
| Preop-LVEF, % | 65.0(61.0, 70.0) | 66.0(65.0, 70.0) | 0.261 |
| HF medications, n (%) | 25 (52.1%) | 2 (13.3) | 0.008 |
| ABCS | 6.0 (6.0, 6.0) | 6.0 (3.0, 6.0) | 0.338 |
| CPB,n (%) | 40 (83.3%) | 14 (93.3%) | 0.587 |

Abbreviations: pre-op, preoperative; LVEF, left ventricular ejection fraction; HF, heart failure; ABCS, Aristotle basic complexity score; CPB, cardiopulmonary bypass;

Appendix 4. Comparison of postoperative peak cardiac biomarker levels in patients stratified by Aristotle basic complexity level and cardiopulmonary bypass status.

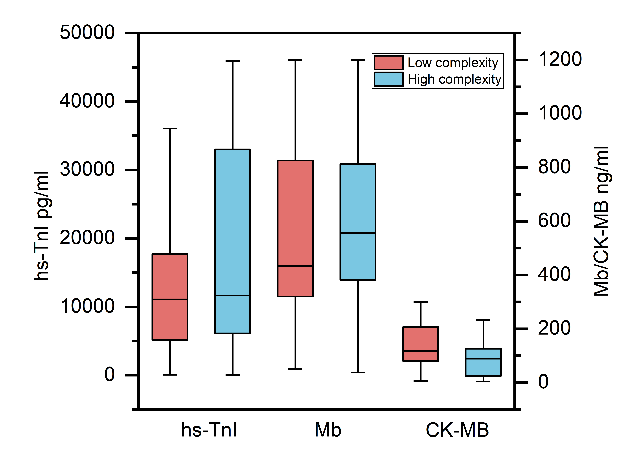

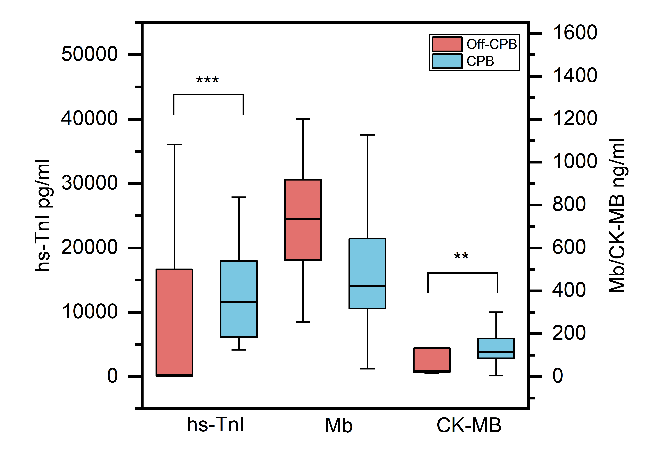


Abbreviations: CPB, cardiopulmonary bypass; hs-TnI, high-sensitivity troponin I; CK-MB, creatine kinase–MB isoenzyme; Mb, myoglobin.

Appendix 5. Friedman test and Bonferroni correction results of LVEF and cardiac biomarkers at different time points. Different superscript letters within a row indicate significant differences between time points after Bonferroni correction (*P* < 0.05). Columns sharing any letter are not significantly different from each other.

|  | Pre-op | POD 1-2 | POD 3-4 | POD5-7 | *P* value |
| --- | --- | --- | --- | --- | --- |
| LVEF (%) | 65.0(60.5, 67.5)^a^ | 58.0(44.5,62.5)^b^ | 59.0(56.5,64.5)^ab^ | 60.0(58.0,61.5)^ab^ | 0.004 |
| hs-TnI (pg/ml) | 17.9(4.8,28.1)^a^ | 12000.7(5186.9, 24571.5)^b^ | 1986.3(847.7,2680.2)^bc^ | 156.3(71.1,361.8)^ac^ | <0.001 |
| CK-MB (ng/ml) | 4.0(2.5,6.5)^a^ | 103.8(70.8,233.6)^b^ | 2.3(1.0,3.3)^a^ | 1.7(1.3,2.9)^a^ | <0.001 |
| Mb (ng/ml) | 25.4(14.1,59.3)^a^ | 356.1(125.3,535.7)^b^ | 16.7(10.0,34.4)^a^ | 18.6(9.0,26.3)^a^ | <0.001 |

Abbreviations: LVEF, left ventricular ejection fraction; hs-TnI, high-sensitivity troponin I; CK-MB, creatine kinase–MB isoenzyme; Mb, myoglobin.
